# Fatigue During Cancer-Related Radiotherapy and Associations with Activities, Work Ability and Quality of Life: Paying Attention to Subgroups more Likely to Experience Fatigue

**DOI:** 10.1177/15347354221138576

**Published:** 2022-11-29

**Authors:** Kristina Tödt, Maria Engström, Magnus Ekström, Anna Efverman

**Affiliations:** 1 University of Gävle, Gävle, Sweden; 2 Skåne University Hospital, Lund, Sweden; 3 Lund University, Lund, Sweden

**Keywords:** fatigue, cancer survivors, radiotherapy, quality of life, activities of daily living, physical activity, work ability, oncology nursing

## Abstract

**Background:**

Having knowledge of which patients are more likely to experience fatigue during radiotherapy and the relationship between fatigue and health-related quality of life (HRQL) is important to improve identification and care of patients experiencing burdensome fatigue.

**Objective:**

To identify subgroups of patients, varying in situational, physiological, and psychological factors, who are more likely to experience fatigue an ordinary week of radiotherapy, and to compare patients experiencing and not experiencing fatigue regarding perceived HRQL and functional performance, that is, daily and physical activity and work ability.

**Methods:**

Cross-sectional study of 457 patients (52% women) undergoing radiotherapy (38% breast, 32% prostate cancer), using self-reported questionnaire data on fatigue, HRQL and functional performance analyzed using multivariable regression models.

**Results:**

Of the 448 patients who answered the fatigue question, 321 (72%) experienced fatigue. Patients reporting any comorbidity or depressed mood were more likely to experience fatigue, relative risk (RR) 1.56 ([95% confidence interval (CI)] 1.13-2.16) and RR 2.57 (CI 1.73-3.83), respectively. Patients with fatigue reported worse HRQL and performed less physical activity, including daily (*P* = .003), vigorous (*P* = .003) and moderate (*P* = .002) activity. Patients with and without fatigue reported 60% versus 40% sickness absence.

**Conclusion:**

Patients with depressed mood or comorbidity were more likely to experience fatigue an ordinary week of radiotherapy than other patients were. Patients experiencing fatigue perceived worse HRQL and performed less daily and physical activity compared to patients not experiencing fatigue. Cancer care practitioners may consider paying extra attention to these subgroups of patients.

## Introduction

Fatigue is one of the most common^1^ and quality-of-life debilitating^2^ symptoms in patients with cancer, though it still seems to be underreported, underdiagnosed, and undertreated during radiotherapy for cancer.^3,4^ Thus, cancer care practitioners in integrative oncology should have access to knowledge needed to identify patients at risk of experiencing fatigue during radiotherapy and understand the relation between fatigue and health-related quality of life (HRQL) as well as functional performance. Cancer-related fatigue refers to “a pervasive, subjective sense of tiredness persisting over time, interferes with activities of daily living, and is not relieved by adequate rest or sleep.”^5^ Around half of patients with cancer receive radiotherapy as part of their cancer therapy. Up to 90% of them have been found to report fatigue.^1,6^ Fatigue typically decreases after radiotherapy,^7-12^ but may be long lasting and remain several years after completed radiotherapy.^13,14^

Various risk factors for fatigue have been identified that are related to the cancer and its therapies or to the individual patient and his/her surrounding context.^15^ The theory of unpleasant symptoms includes 3 major concepts: “influencing factors,” “symptoms,” and “performance.”^16^ This theory offers a conceptual framework that might improve health care professionals’ understanding of the relationship between factors influencing the experience of a symptom, that is, risk factors, and the consequences of the symptom/symptoms for performance,^16^ and HRQL.^17^ Performance, including, for example, daily and physical activity and work, is thought to have a reciprocal relationship with influencing factors and symptom experience. The theory proposes influencing factors that are physiological (eg, clinical data), situational (eg, demographics, lifestyle factors), and psychological (eg, mental state or mood).^16^ Previous studies have demonstrated that different physiological factors—such as type of cancer,^6^ stage of disease,^9,18,19^ concomitant chemotherapy,^11,18-20^—and situational factors—such as higher educational level, and living alone^8,10^—were related to fatigue during radiotherapy. However, there are inconsistent findings regarding some factors’ relationship with fatigue. For example, neither age nor gender predicted fatigue during radiotherapy for cancer,^6^ while younger age was associated with worse fatigue in other studies.^8,9,11^ Even if cancer type, stage of disease and treatment may give rise to fatigue, other factors may be of greater importance to the experience.^15^ The psychological influencing factor depressed mood may plausibly influence symptom experience,^16^ an effect observed by others regarding cancer-related fatigue.^15,21^ In addition, several other symptoms commonly co-occur with fatigue during a variety of cancer therapies,^22^ radiotherapy included.^2,22,23^ The concurrence of symptoms is thought to result in a multiplicative experience.^16^

Fatigue was associated with poor perceived quality of life in patients undergoing radiotherapy^5,10,24^ and was one of the most predictive symptoms for perceived overall quality of life at the completion of radiotherapy.^2^ However, in one study, despite increasing fatigue during radiotherapy, HRQL remained stable,^25^ indicating the need for further studies on the relationship between fatigue and HRQL during radiotherapy.

The search for safe and effective pharmacological treatments for fatigue continues.^26^ However, in patient-centered integrative oncology, complementary therapies, and lifestyle modifications, such as physical activity, are used alongside pharmacological and other kinds of conventional treatments.^27^ Regarding the reciprocal relation between fatigue and performance,^16^ fatigue was observed to be a barrier to practicing physical activity during various cancer therapies.^28^ At the same time, physical activity has been shown to reduce fatigue.^1,29,30^ Engaging in physical activity during radiotherapy is thus advocated.^5,31^ If evidence from physical activity efficacy studies had been successfully implemented within routine radiotherapy care, we would expect patients experiencing fatigue today to practice more physical activity than has been seen in previous studies.^32^ Although cancer therapies in general decreased functional performance, that is, capacity to perform daily activities,^33^ there are few studies describing the relationship between symptoms and daily activities specifically during radiotherapy.^34^ Fatigue during radiotherapy may also interfere with other aspects of functional performance,^16^ such as work ability^35^ and thus contribute to sickness absence, but the findings are not clear.^8,9,22^

Although studies have observed fatigue during a variety of cancer therapies,^1,18^ fewer studies have looked at patients specifically undergoing radiotherapy, being treated in a routine cancer care setting and not participating in randomized controlled studies evaluating a certain radiotherapy technique.^6,22^ Previous results are conflicting regarding subgroups of patients who are more likely to experience fatigue than others.^6,8,9,11,20^ Further, the potential relationship between fatigue and HRQL,^2,24,25^ as well as functional performance, during radiotherapy, that is, daily activities^34^ and work ability^8,9,22^ is still underreported.

The present study focused on patients who were receiving radiotherapy for cancer in a routine care setting. It aimed to identify subgroups of patients, varying in situational, physiological, and psychological factors, who are more likely to experience fatigue an ordinary week of radiotherapy. It also aimed to compare patients experiencing and not experiencing fatigue regarding perceived HRQL and functional performance, that is, daily and physical activity and work ability.

## Methods

### Design and Setting

We conducted this cross-sectional study in a routine care setting at the radiotherapy departments of 4 oncology clinics in southern, western, and eastern Sweden. Oncology care in Sweden is taxpayer funded and out-of-pocket fees are low and regulated by law. According to the Swedish social insurance system, the patients’ physicians prescribe sick leave in not retired patients in case of reduced working ability (25%, 50%,and 75%, or 100% lack of work ability), irrespective whether the patients are employed or not employed (job seekers, participating in job seeking, and job practice activities), or students. A pilot study showed that the study questionnaire and procedures were feasible.^36^ The study complies with the declaration of Helsinki—Ethical principles for medical research involving human subjects and the Regional Ethical Committee approved the study (Linköping 2015 /101-31). All participants gave their informed written consent.

### Sample

Within the study period June to December 2016, we randomly selected one study day at each radiotherapy clinic. Radiotherapy nurses who were coordinating the study at each radiotherapy department screened all patients scheduled for radiotherapy for the study criteria and provided them with verbal and written study information the day before the study day (ie, the day for data collection). Then they asked for the patients’ informed consent on the study day. Inclusion criteria were patients undergoing fractioned radiotherapy for cancer regardless of radiotherapy field, cancer diagnosis or disease stage (curative and palliative cancer), who were >18 years old and were judged to have the physical, mental, and linguistic capacity needed to give their informed consent. Exclusion criteria were patients who were receiving their very first or single radiotherapy fraction on the study day (ie, patients receiving one-fraction palliative radiotherapy were excluded).

### Data Collection and Procedure

When the patients arrived to receive their ordinary radiotherapy session on the study day, the routine care radiotherapy nurses gave a study questionnaire to the included patients and stressed that they could not read the patients’ responses; only the study evaluator had access to the response data. The patients completed the questionnaire at home or at the patient hotel or ward unit, either in writing, using paper and pen, or on the study’s confidential web-based study database. They were free choose the place and method. The patients returned paper questionnaires in designated questionnaire response boxes at the radiotherapy department, or by post using pre-paid envelopes. If the questionnaire was not returned after about 2 weeks, the radiotherapy nurses coordinating the study reminded the non-respondents by calling them. The study-specific questionnaire items are presented in Appendix 1.

### The Study Questionnaire

#### Situational, physiological, and psychological data

Situational data^16^ included educational level, occupational status, marital status, and native country. As one situational lifestyle variable, the questionnaire asked for the level of physical activity during a regular week the year prior to the cancer diagnosis. The patients reported the level of physical activity by choosing 1 of 7 categorized intervals of minutes for moderate physical activity and 1 of sex for vigorous activity.^37^ The physiological data considered were age, sex, other cancer treatment in addition to external radiotherapy (yes/no), extensive disease (yes/no) and self-reported presence of other diseases (comorbidity) (yes/no). Physiological data obtained from the patients’ medical records included data on cancer diagnosis, irradiated field, and dose of radiotherapy. Presence of anxious and depressed mood were considered psychological influencing factors on fatigue and were assessed on an 8-point scale between 0 = never and 7 = all the time.^38^

#### Assessment of fatigue and other symptoms

Fatigue was assessed using one study-specific question, developed according to clinimetric methodology ^39^: “Have you felt any extreme tiredness that has been difficult to relieve by resting, during the past week.” Responses were given on an 11-point scale (0 = never, not present and 1-10 = presence of fatigue with 10 = fatigue present all the time) (Appendix 1). Clinimetrics concerns single-item rating scales and other expressions that are used to feasibly, validly, and reliably describe or measure symptoms and other distinctly clinical phenomena in line with how the target population describes the symptoms or phenomena.^40^ Other cancer-related symptoms were assessed using the Swedish version^41^ of The Memorial Symptom Assessment Scale (MSAS).^42^

#### Assessment of HRQL

The study questionnaire assessed HRQL using the valid, reliable and generic EuroQol 5 Dimensions-3 levels (EQ5D-L3)^43^ Swedish version,^44^ widely used among patients with cancer.^45^ EQ5D-L3 comprises 5 dimensions: mobility, self-care, usual activities, pain/discomfort and anxiety/depression, rated as: 1; no problems, 2; some problems, 3; extreme problems. The grading contributes to a EQ5D-L3 index score (−1 to 1, complete health).^43^ EQ5D-L3 also includes the vertical visual analog scale, EQ-VAS, measuring overall health (0, worst imaginable health, to 100, best imaginable health).^43,44^ Further, the Swedish version^46^ of the valid and reliable Functional Assessment of Cancer Therapy-General (FACT-G)^47^ assessed HRQL. The 27-item FACT-G covers four domains: physical wellbeing (7 items), social wellbeing (7 items), emotional wellbeing (6 items), and functional wellbeing (7 items). Using FACT-G, the evaluator calculated sub-scores and a total score. Higher scores represent better HRQL.

#### Assessment of functional performance

The patients assessed their capacity in daily activities the preceding week using 7 graded statements ranging from “I managed all my daily activities” to “I did not manage any of my daily activities” (Appendix 1).^34^ Physical activity^48^ the preceding week was assessed in the same way as physical activity prior to cancer, described above. We used the patients’ self-reported percentage of sickness absence (0%, 25%, 50%, 75%, or 100% sick leave) as a proxy for work ability.^35^ Patients on disability pension were accordingly classified as having 100% sickness absence. Students, the unemployed and other patients were accordingly classified as having 0% sickness absence if they were not on sickness leave.

### Statistical Analysis

Descriptive data were presented as frequencies (n), %, mean (m) with standard deviation (±SD), and median (md) with 25th to 75th percentile (IQR). The fatigue rating was correlated with the accumulated radiotherapy dose using Pearson’s correlation coefficient, *r*. Based on their response to the fatigue question, we categorized patients into 2 groups: “No fatigue” (stated score 0) and “Experience fatigue” (stated score 1-10). Fischer’s exact test compared subgroups of patients with different situational, physiological and psychological characteristics,^16^ presented as relative risks (RR) of experiencing fatigue, with 95% Confidence Interval. The reference category was defined as the category with the lowest proportion experiencing fatigue. We selected variables, resulting in *P* < .20 according to the univariable analysis (all variables seen in Table 2), to be further explored regarding their potential role in explaining the variation in presence of fatigue or lack of fatigue using a multivariable generalized linear model, and we generated relative risks using binomial distribution and cloglog link.

We compared patients with and without fatigue using student’s *t*-test for HRQL (EQ5D-3L index score, EQ-VAS, FACT-G, analyzed as a continuous variable^47^ and work ability (ie, % sickness absence), and Mann Whitney *U*-test for functional performance (daily and physical activity within the preceding week). To analyze whether differences in HRQL and functional performance were still valid after adjustment for the situational, psychological, and functional performance variables that we (based on interpreting the generalized linear model mentioned above) knew differed between patients with and without fatigue and that theoretically also may impact our outcome variables, multiple univariate analyses of covariance (ANCOVA)^49^ were performed with EQ5D-3L index score, EQ-VAS, FACT-G total and sub-scores, daily and physical activity (moderate and vigorous) and work ability as dependent variables and experience of fatigue yes/no, comorbidity yes/no and depressed mood (score 1-7 included in the analyses as a covariate) as independent variables. Model assumptions in the univariate analyses were checked using Levene’s test and standard graphical procedures.

Statistical significance was defined as *P* < .05 (two-tailed). All analyses were performed in IBM SPSS statistics version 27.0 (IBM Corp, Armonk NY, USA).

## Results

### The Participating Patients

**Figure 1.**
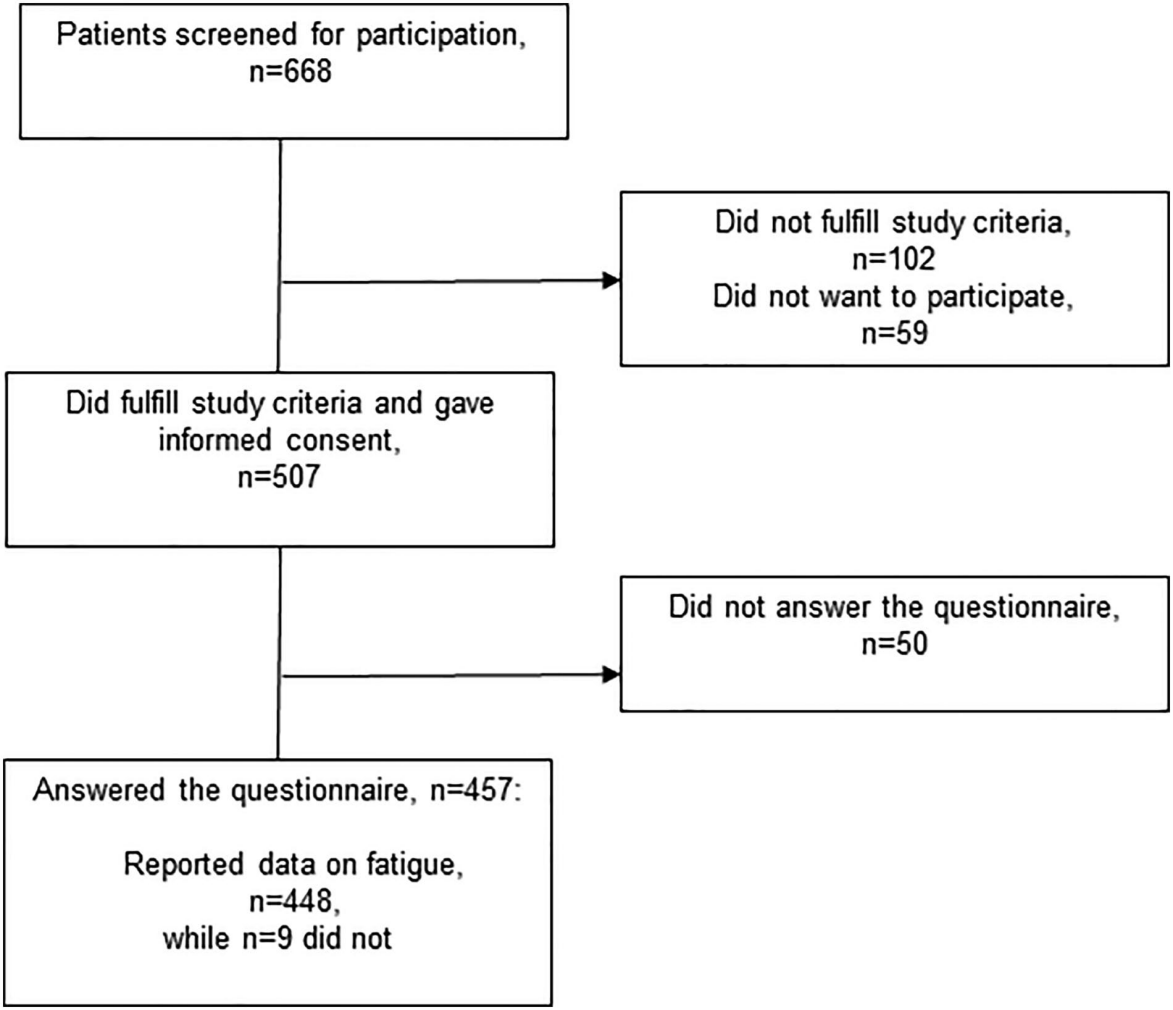
Flowchart of the screened, included and responding patients undergoing cancer-related radiotherapy.

Of the 668 patients screened for inclusion, 507 met the study criteria and wanted to participate. Of these 507, 457 returned their data (response rate 90%) (Figure 1). Approximately half were men (n = 221; 48%), 182 (40%) were not retired, and patients were mostly treated for breast (n = 171; 38%) or prostate cancer (n = 145; 32%) (Table 1).

**Table 1.** Demographic and Clinical Characteristics.

Variable	Total study group of patients receiving cancer-related radiotherapy, N = 457
Sex, n (%), n = 457	
Women	236 (52)
Men	221 (48)
Age in years, *m* ± SD, range, n = 454	65 ± 12, 24-90
Highest education level, *n* (%), n = 448	
Elementary school	76 (17)
Secondary school	100 (22)
Vocational school	118 (26)
University	154 (34)
Occupational status, *n* (%), n = 455	
Employed^a^	144 (32)
Retired	273 (60)
Not employed, student and other	22 (5)
On disability	16 (4)
Marital status, *n* (%), n = 457	
Married or co-habiting	341 (75)
Living alone; have a partner	23 (5)
Living alone; single or widow/widower	93 (20)
Born in the country for the study, *n* (%), n = 453	
Yes, born in Sweden	401 (88)
Cancer type, *n* (%), n = 450	
Breast cancer	171 (38)
Prostate cancer	145 (32)
Head/neck and brain cancer	57 (13)
Colon, rectal, anal, and gynecological cancer	43 (10)
Lymphoma and cancer in supporting tissues	19 (4)
Lung cancer	15 (3)
Irradiated field, *n* (%), n = 449	
Breast	165 (37)
Prostate or bladder	144 (32)
Head or neck	63 (14)
Abdomen or pelvis	47 (10)
Thorax, mediastinum, lung	27 (6)
Other, eg, extremities	3 (1)
Radiotherapy characteristics, mean ± SD	
Gy per fraction, n = 449	2.7 ± 0.55
Accumulated dose (Gy), n = 450	33.3 ± 17.0
Cancer therapies other than external radiotherapy, *n* (%)	
Tumor surgery, n = 450	278 (62)
Chemotherapy, n = 442	166 (38)
Hormone therapy, n = 441	159 (36)
Internal radiotherapy, n = 442	52 (12)
Extensive disease, *n* (%), n = 409	
Yes/no	61 (15)/348 (85)
Comorbidity, *n* (%), n = 424	
Yes/no	187 (44)/237 (56)
Number of symptoms,^b^ median, IQR, *n* (%), n = 408	8, 4-14
4 symptoms or less	109 (27)
5 symptoms or more	299 (73)
Moderate physical activity prior to cancer, *n* (%), n = 453	
0-150 min	270 (60)
>150^c^ min	183 (40)
Vigorous physical activity prior to cancer, *n* (%), n = 447	
0-90 min	333 (74)
>90^d^ min	114 (26)

n (number) and proportion (%) of participants are presented except as indicated. *n* of patients delivering data is presented.

Abbreviations: ±SD, 1 standard deviation; Gy, gray.

a Of these, 101 were partly or fulltime on sick leave.

b Number of occurring symptoms the past week according to the Memorial Symptom Assessment Scale, 0 to 31 possible symptoms, the symptom lack of energy excluded. The 25th percentile was used as a cut-off when categorizing the variable.

c Guidelines recommend at least 150 minutes physical activity a week.^30^

d The 75th percentile was used as a cut-off when categorizing the variable.

### Experience of Fatigue and Other Symptoms

**Figure 2.**
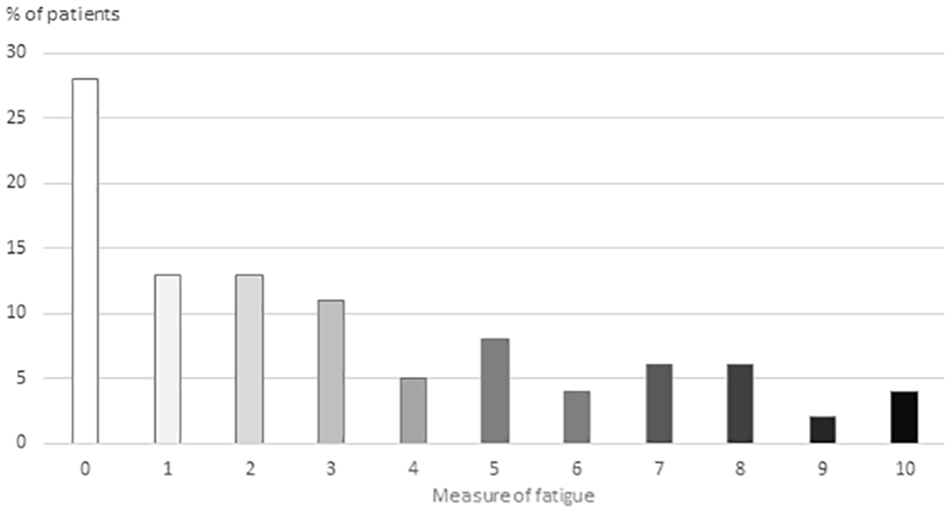
Percentage of patients with different ratings of “an extreme tiredness that has been difficult to relieve by resting, during the past week,” that is, fatigue, 0 = no fatigue to 10 = fatigue all the time. n = 448 delivered data.

Of the 448 patients who answered the fatigue question, 321 (72%) experienced fatigue, with a median rating of 4 (IQR 3-7) (Figure 2). Higher accumulated dose of radiotherapy was not statistically significantly (*P* = .405) correlated with more fatigue (*r* = .040). The patients reported a median of 8 (IQR 4-14) concurrent symptoms on MSAS, the symptom “lack of energy” excluded (md 8, IQR 5-15, if lack of energy was included). On the MSAS, “lack of energy” was reported by n = 301 (68%) of patients. Other common symptoms, reported by >40% of patients, were “problems with sexual interest or activity,” “feeling drowsy,” “difficulty sleeping,” “pain,” “sweats,” “worrying,” “feeling sad,” and “dry mouth” (Appendix 2).

### Subgroups of Patients more Likely than Others to Experience Fatigue

According to the multivariable analysis, patients with presence of depressed mood and patients with self-reported comorbidity were more likely to experience fatigue compared to other patients (Table 2).

**Table 2.** Frequency of Experience of Fatigue in Subgroups of Patients Undergoing Radiotherapy with a Variety of Situational, Physiological and Psychological Factors.

Variable, *n* (%)	No fatigue, n = 127	Fatigue, n = 321	Relative risk, 95% CI, *P*-value univariable analyses (crude models), n = 448	Relative risk, 95% CI, *P-*value multivariable analysis (adjusted model), n = 345
Situational factors				
Highest educational level	*n* = 124	*n* = 315		
Elementary school	24 (32)	50 (68)	Ref.	Ref.
Secondary school	23 (23)	77 (77)	1.14 (0.94-1.38), .172	1.17 (0.71-1.92), .544
Vocational school	32 (28)	83 (72)	1.07 (0.88-1.30), .517	0.99 (0.62-1.61), .985
University	45 (30)	105 (70)	1.04 (0.86-1.25), .759	0.87 (0.54-1.39), .575
Occupational status	n = 127	n = 319		
Employed, student, not employed and other	21 (32)	44 (68)	Ref.	Ref.
Any level of sickness absence	23 (19)	101 (81)	1.20 (1.0-1.45), .046	1.28 (0.76-2.15), .353
Retired	83 (32)	174 (68)	1.00 (0.83-1.20), 1.00	0.82 (0.49-1.36), .435
Marital status	*n* = 127	n = 321		
Married or co-habiting, have a partner	110 (31)	247 (69)	Ref.	Ref.
Living alone; single or widow/widower	17 (19)	74 (81)	1.17 (1.04-1.33), .026	1.20 (0.81-1.77), .364
Born in the country for the study	*n* = 125	*n* = 319		NA
Yes, born in Sweden	110 (29)	284 (72)	1.03 (0.85-1.25), .741	
No	15 (30)	35 (70)	Ref.	
Moderate physical activity prior cancer	n = 127	n = 320		
0-150 min	63 (24)	205 (76)	1.19 (1.05-1.35), .005	1.24 (0.90-1.69), .184
>150^b^ min	64 (36)	115 (64)	Ref.	Ref.
Physiological factors				
Sex, *n* (%)	n = 127	n = 321		
Men	65 (30)	151 (70)	Ref.	NA
Women	62 (27)	170 (73)	1.05 (0.93-1.18), .463	
Age^a^	n = 124	n = 320		
18-44 year	5 (17)	24 (83)	1.21 (1.00-1.47), .128	0.93 (0.44-1.97), .849
45-69 year	68 (24)	186 (73)	1.07 (0.94-1.22), .316	1.0 (0.67-1.48), .986
≥70 year	51 (32)	110 (68)	Ref.	Ref.
Cancer type	*n* = 125	*n* = 315		NA^e^
Lymphoma and cancer in supporting tissues	7 (41)	10 (59)	Ref.	
Breast cancer	45 (27)	123 (73)	1.25 (0.83-1.87), .257	
Prostate cancer	41(29)	100 (71)	1.21 (0.80-1.82), .402	
Head/neck and brain cancer	13 (23)	43 (77)	1.31 (0.86-1.99), .213	
Colon, rectal, anal, gynecological cancer	17 (40)	26 (60)	1.03 (0.65-1.64), 1.0	
Lung cancer	2 (13)	13 (87)	1.47 (0.94-2.30), .122	
Irradiated field	*n* = 124	*n* = 315		
Abdomen or pelvis and other eg, extremities	20 (40)	30 (60)	Ref.	Ref.
Breast	44 (27)	118 (73)	1.21 (0.95-1.55), .112	1.27 (0.73-2.23), .396
Prostate or bladder	41 (29)	99 (71)	1.18 (0.92-1.51), .216	1.57 (0.85-2.91), .150
Head or neck	15 (24)	47 (76)	1.26 (0.97-1.65), 0.101	1.16 (0.62-2.17), .633
Thorax, mediastinum, lung	4 (16)	21 (84)	1.40 (1.05-1.86), .040	1.85 (0.81-4.24), .147
Accumulated dose				
0-43 Gy	95 (30)	222 (70)	Ref.	Ref.
≥44^c^ Gy (=75th percentile)	28 (23)	94 (77)	1.00 (0.97-1.24), .120	1.22 (0.85-1.75), .281
Cancer therapies other than external radiotherapy				
Tumor surgery	*n* = 122	*n* = 319		
No	43 (25)	126 (75)	1.05 (0.94-1.18), .444	NA
Yes	79 (29)	193 (71)	Ref.	
Chemotherapy	*n* = 119	*n* = 314		
No	83 (31)	185 (69)	Ref.	Ref.
Yes	36 (22)	129 (78)	1.13 (1.01-1.26), .046	1.07 (0.71-1.60), .755
Hormone therapy	*n* = 116	*n* = 316		
No	79 (29)	197 (71)	Ref.	NA
Yes	37 (24)	119 (76)	1.07 (0.95-1.20), .309	
Internal radiotherapy	*n* = 117	*n* = 316		
No	104 (27)	279 (73)	Ref. 1.0	NA
Yes	13 (26)	37 (74)	1.02 (0.85-1.21), 1.0	
Extensive disease	*n* = 117	*n* = 292		NA
No	103 (30)	245 (70)	Ref.	
Yes	14 (23)	47 (77)	1.09 (0.94-1.28), .357	
Comorbidity	*n* = 123	*n* = 294		
No	85 (36)	150 (64)	Ref.	Ref.
Yes	38 (21)	144 (79)	1.24 (1.10-1.40), <.001	1.56 (1.13-2.16), .007
Number of symptoms^d^	*n* = 118	*n* = 285		
4 symptoms or less (=25th percentile)	57 (52)	52 (48)	Ref.	Ref.
5 symptoms or more	61 (21)	233 (80)	1.66 (1.35-2.04), <.001	1.25 (0.84-1.87), .264
Psychological factors				
Depressed mood	n = 127	n = 321		
No	96 (50)	96 (50)	Ref.	Ref.
Yes	31 (12)	225 (88)	1.76 (1.52-2.04), <0.001	2.57 (1.73-3.83), <.001
Anxious mood	*n* = 127	*n* = 321		
No	109 (39)	171 (61)	Ref.	Ref.
Yes	18 (11)	150 (89)	1.46 (1.31-1.63), <.001	1.17 (0.79-1.74), .427

*n* (number) and proportion (%) of participants are presented, *n* of patients delivering data is presented in case of missing data.

Abbreviations: Ref., reference category, relative risk 1.0; the category with the lowest proportion reporting fatigue; CI, 95% confidence interval.

a The age groups are based on Statistics Sweden’s population age groups.

b Guidelines recommend at least 150 minutes physical activity a week.^30^

c The 75th percentile was used as a cut-off when categorizing the variable, that is, the patients who had received >44 Gy were at the last part of their radiotherapy period. Number of symptoms on MSAS ≤ 4 versus >4 symptoms (cut-off by the 25th percentile) and presence of anxious and depressed mood score ≥1 versus not presence score 0.

d Summed n of symptoms within the past week on the Memorial Symptom Assessment Scale, 0 to 32 possible symptoms, the symptom lack of energy excluded. NA = Not Applicable; Not included in the multivariable analysis because *P* was not <.20 in the univariable analysis.

e Due to low *n* when combined with irradiated field in the multivariable analysis, irradiated field was chosen.

### Fatigue and HRQL

Patients experiencing fatigue reported worse EQ5D-index and EQ-VAS, m 0.727 ± SD 0.24 and m 64 ± SD 21, versus 0.893 ± SD 0.15 and 81 ± SD 16, in patients not experiencing fatigue (Table 3). The patients experiencing fatigue, compared to non-fatigued patients, rated worse HRQL on the FACT-G total score and the 4 sub-scores (Table 3). The differences in HRQL between patients experiencing and not experiencing fatigue remained even when adjusted for comorbidity and depressed mood, except for the emotional and social wellbeing dimensions of FACT-G (Table 3).

**Table 3.** Health-Related Quality of Life and Functional Performance in Patients Undergoing Cancer-Related Radiotherapy with and Without Experiencing of Fatigue.

Variable	No fatigue n = 127	Experience fatigue n = 321	Mean difference and (95% CI), *P-*value	Adjusted^b^ mean difference and (95% CI), *P-*value
Health-related quality of life, m (±SD)				
EQ-5D index score, n = 448	0.893 ± 0.152	0.727 ± 0.235	0.166 (0.122-0.210), <.001	0.067 (0.024-0.110), .002
EQ-5D VAS 0-100, n = 442	81 ± 16	64 ± 21	16.6 (12.9-20.3), <.001	8.12 (4.02-12.22), <.001
FACT-G, total score, n = 433	92 ± 10.6	77.7 ± 14.9	14.3 (11.7-16.8), <.001	6.25 (3.75-8.74), <.001
Physical well-being sub-score, n = 443	25.7 ± 3.0	20.6 ± 5.4	5.1 (4.1-6.1), <.001	2.8 (1.9-3.8), <.001
Social/family well being-sub-score, n = 441	24.3 ± 4.8	22.8 ± 4.8	1.5 (0.5-2.5), .003	0.33 (-0.71 to 1.36), .539
Emotional well being-sub-score, n = 446	21.1 ± 2.9	18.2 ± 4.5	2.9 (2.2-3.6), <.001	0.64 (-0.11 to 1.32), .097
Functional well being-sub-score, n = 447	20.8 ± 4.8	16.0 ± 5.3	4.8 (3.8-5.9), <.001	2.45 (1.43-3.48), <.001.
Functional performance, m (±SD)				
Daily activities, n = 441	6.6 ± 1.1	5.7 ± 1.5	0.89 (0.59-1.18), <.001	0.48 (0.17-0.78), .003
Vigorous physical activity, n = 442	2.6 ± 1.7	2.0 ± 1.3	0.61 (0.32-0.91), <.001	0.50 (0.18-0.83), .003
Moderate physical activity, n = 445	4.7 ± 1.9)	3.7 ± 1.8	0.94 (0.57-1.31), <.001	0.65 (0.24-1.06), .002
Work ability^a^, n = 181	n = 42	n = 139		
Sickness absence in % sick leave	39.9 ± 45.2	62.0 ± 46.3	22.2 (6.2-38.2), .007	13.0 (−4.5 to 30.4), .146

m, mean; ±1 SD, standard deviation, is presented. *n* delivering data is presented. Physical Well-Being, Social/family Well-Being, Emotional Well-Being, Functional Well-Being, possible sub-scores range 0 to 28, except for Emotional Well-Being 0 to 24. Daily activities score 1 to 7, moderate physical activity score 1 to 7, vigorous physical activity score 1 to 6. Higher values indicate better HRQL and functional performance.

Abbreviations: CI, 95% confidence interval; EQ-5D, EuroQol 5-dimension; VAS, Visual Analog Scale; FACT-G, Functional Assessment of Cancer Therapy-General, possible total score ranges 0 to 108.

a Work ability, that is, sickness absence, is presented for all not retired patients.

b Adjusted for comorbidity yes/no, and depression 1 to 7.

### Fatigue and Functional Performance

#### Daily and physical activity

**Figure 3.**
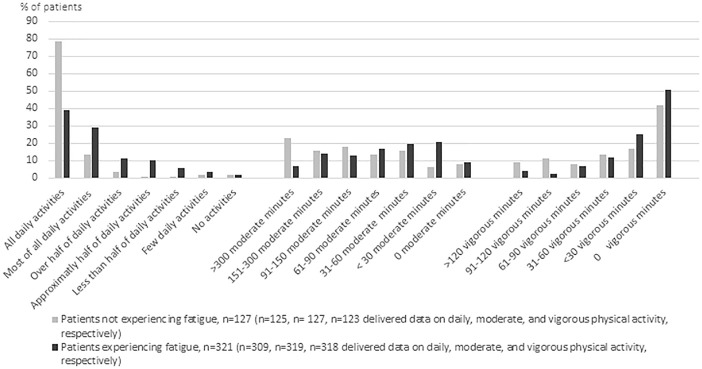
Percentage of patients managing different amounts of daily activities and minutes practiced in moderate and vigorous physical activity in the 321 patients experiencing fatigue (fatigue score 1-10) compared to the 127 patients not experiencing fatigue (fatigue score 0).

The patients not experiencing fatigue managed larger amounts of their daily activities compared to patients experiencing fatigue (*P* < .001, adjusted univariate analysis of variance *P* = .003). For example, 80% of patients not experiencing fatigue managed all their daily activities compared to 40% of patients experiencing fatigue (Figure 3). The patients not experiencing fatigue also spent more minutes per week engaged in moderate (*P* < .001) and vigorous (*P* = .002) physical activity during the week preceding radiotherapy compared to the patients experiencing fatigue. Of the patients without versus with fatigue, 39% versus 21% practiced >150 minutes of moderate intensity physical activity (Figure 3). The differences remained after adjusting for comorbidity and depressed mood (Table 3).

#### Work ability

For those 182 patients eligible to analyze (ie, not retired), 68 (37%) reported no sickness absence and 114 (63%) reported some level of sickness absence, with the majority (n = 92, 81%) reporting 100% sickness absence. The unadjusted higher level of sickness absence (mean difference 22.2% sickness absence, CI 6.2-38.2) in patients who experienced fatigue compared to patients without fatigue did not remain after adjusting for comorbidity and depressed mood (Table 3). Additional analysis, in which depressed mood was included as a fixed factor, showed that presence of depressed mood was associated with higher level of sickness absence (adjusted mean difference 24.7%, CI 9.1-40.4), while experiences of fatigue and comorbidity were not.

## Discussion

The main findings were that approximately 3 quarters of patients experienced fatigue within an ordinary week of radiotherapy for cancer and patients who reported having depressed mood or comorbidities were more likely to experience fatigue than other patients were. Patients experiencing fatigue had poorer HRQL and performed less daily and physical activity.

After receiving an accumulated dose of median 32 Gy, that is, approximately 2 or more weeks of radiotherapy, as many as 72% of the patients had experienced fatigue within the past week of radiotherapy. The proportion of patients experiencing fatigue did not increase over the course of radiotherapy. That is, those patients who had received a higher accumulated dose of radiotherapy at the time point for the study were no more likely to experience fatigue than those who had received a lower accumulated dose. In line with this, the accumulated dose was not correlated with more fatigue. Also, previous studies regarding patients undergoing radiotherapy reported rather similar proportions of patients experiencing fatigue irrespective of the time point observed; 68%, 78%,^6^ and 81%^22^ experienced fatigue in the second week, fifth week, and at the completion of radiotherapy (mostly after 5-6 weeks), respectively. The finding that patients with depressed mood and/or any comorbidity were more likely to experience fatigue is in accordance with results from previous studies^9,21^ of 73 and 210 women, respectively, undergoing radiotherapy for breast cancer. Depressed mood was associated with fatigue even 6^14^ and 10 years^13^ after cancer therapy. Our findings indicate the importance of identifying patients with depressed mood and fatigue already during the radiotherapy period to avoid long-term suffering. Repeated symptom assessment during radiotherapy reduced the patients’ fatigue.^50^ In contrast to previous studies showing that the physiological factors^16^ cancer type and stage^6,9,18,19^ or prior or concomitant chemotherapy^11,18,20^ were associated with fatigue, our study did not reveal such associations. Nor was younger age, female sex, higher educational level, and living alone^6,8,10,11^ related to worse fatigue when the variables presence of depressed mood and comorbidity were included in the multivariable regression model together with the other physiological, situational, and psychological influencing factors. Fatigue in patients undergoing radiotherapy is probably induced by several sources beside the radiotherapy and the cancer illness,^15^ some of which are possible targets for rehabilitation and support from cancer care practitioners.

Fatigue was associated with worse HRQL, even after adjustment for depressed mood and comorbidity, which is consistent with other studies.^2,10,24^ Fatigue was the symptom with the strongest negative relation to quality of life in 1224 patients with breast cancer.^2^ The differences in FACT-G total and sub-scores between patients experiencing and not experiencing fatigue may be considered moderate.^51^ This finding indicates that fatigue still needs to be identified and treated to avoid worsening HRQL.

The finding that patients experiencing fatigue performed less daily and physical activity, of both moderate and vigorous intensity, than did patients not experiencing fatigue supports the applicability of the theory of unpleasant symptoms^16^ in the target population. Despite the strong scientific evidence for the effect of physical activity on cancer-related fatigue,^1,5,29-31^ our findings indicate that guidelines proposing physical activity^30^ in patients undergoing radiotherapy have not been successfully implemented in routine care. In fact, patients seem to need more and individualized information about the effects of physical activity on fatigue.^28,52^ The recent research showing that low to moderately intensive activities had similar fatigue-reducing effects as vigorous intensity activities^53^ indicates that well-prepared home-based exercise, incorporated into daily activities,^54^ may be a way to increase beneficial daily and physical activity. Of persons still experiencing fatigue up to 3 years after various cancer therapies, 91% reported moderate to severe functional disability, which was 3 times more than in patients not experiencing fatigue.^14^ These findings and those from the present study highlight the need for encouragement of patients experiencing fatigue, to engage in beneficial daily and physical activity and avoid harmful inactivity.

Fatigue was not associated with more sickness absence in the 181 patients in working ages (ie, the employed, not employed [job seeking], or studying patients), after adjusting for presence of depressed mood and comorbidity. An alternative way of analyzing the association between fatigue and sickness absence would have been to include only the 144 employed patients in the analysis (n = 143 delivered fatigue data). However, this would not have changed our interpretation; there was no statistical difference in sickness absence between the 110 patients experiencing fatigue compared to the 33 patients without fatigue, mean difference 13.1% and CI -30.8 to 4.6 (the figures are not presented in the results, just for discussion). Irrespective of the way of analyzing the association between fatigue and sickness absence, the presence of depressed mood seemed to explain more of the variation in sickness absence than did fatigue per se. Symptoms and illnesses often co-occur.^16^ A recent longitudinal study among cancer survivors demonstrated that comorbidity and mental disorders, depression among others, predicted future sickness absence.^55^ Work ability covers more aspects than sickness absence.^35^ For example, we do not know whether patients experiencing fatigue perceive worse work performance than patients not experiencing fatigue. The daily activity most frequently affected by symptoms during radiotherapy, fatigue included, was work, according to a previous study (n = 903 patients). More than half reported that work was affected by their symptoms.^22^ Receiving support (eg, opportunities for adjustments at work) in continuing work during cancer therapy, to the extent that it seems possible, may benefit a variety of health, social and economic aspects of life among cancer survivors.^56^

We reviewed our methodology in line with the hierarchical step model for causation of bias.^57^ Regarding confounding, the theory of unpleasant symptoms^16^ guided the selection of independent variables that possibly influence the variance in experiencing fatigue. We included situational demographic and lifestyle (ie, physical activity level prior to cancer) variables and psychological influencing variables in the multivariable analysis to identify subgroups of patients more vulnerable to fatigue. Thus, by adjusting for variables related to greater likelihood of fatigue, HRQL and performance, we reduced the risk of bias due to confounding variables. Regarding misrepresentation, one strength is that 90% of the included patients responded to the study questionnaire. One reason for this may be that we had thoroughly tested the feasibility of the study questionnaire in a pilot study^36^ and adopted feasible procedures regarding the information, inclusion, and data collection procedure.^34,36^ Regarding internal attrition, approximately 10 patients did not return data for the different items of the study questionnaire. Of 457 participants, 448 returned their fatigue data, and we thus interpret the 2.0% missing data to be negligible. Due to the cross-sectional design, findings pertain to associations and causality cannot be inferred. However, this design allowed us to collect data with varying length of follow-up time in relation to accumulated length of the radiotherapy period, thus enabling us to detect the trajectory of fatigue and the functional and HRQL-related variables without the measurement-induced bias that often occurs when making repeated measurements. The single-item fatigue question was developed and validated according to clinimetric methodology.^39,40^ Based on the experiences of patients from the target population, the fatigue question captured the nature of the cancer-related fatigue, that is, a persistent experience that could not be alleviated through rest and covered the frequency of this fatigue in one single question. Adopting clinimetric study-specific questions is a highly established data collection method in cancer care research.^34,36,39,58^ Answers to our single-item fatigue question moderately correlated^59^ with other ways to measure fatigue, for example the item “I have lack of energy” within FACT-G^47^ and severity grading of “lack of energy” within MSAS^42^ (Spearman’s rank correlation, *r_s_* = −.688 and *r_s_* = .582 respectively, figures are not presented in the result, just for the discussion). This indicates satisfactory content validity. Physical activity was self-reported using valid questions.^37^ Adopting objective accelerometer measurements could have entailed the risk of attrition-related bias and excessive burden on patients. There may be other, not-measured, lifestyle factors of relevance to fatigue, such as body mass index.^9^ Regarding the last step in the hierarchical step model,^57^ we categorized patients based on experiencing any fatigue, irrespective of level of fatigue, compared to experiencing no fatigue at all, because in a previous study, increased levels of fatigue over time did not worsen HRQL.^25^ We are greatly aware that some of the variables that were treated as continuous variables^51^ are ordinal variables. However, in line with others,^51^ we consider the risk of bias being induced by this choice to be low. We studied a large sample of patients with a variety of cancer types undergoing radiotherapy in a routine care setting, selected using few exclusion criteria, which strengthens the external validity. However, the exclusion of patients who were physically or mentally too frail to give their informed consent naturally reduces the generalizability of the findings to very frail patients.

To conclude, approximately 3 quarters of patients experienced fatigue during an ordinary week of radiotherapy for cancer and patients with depressed mood or comorbidity were more likely to experience fatigue. Patients experiencing fatigue perceived poorer HRQL, and performed less daily and physical activity compared to patients not experiencing fatigue. This implies that cancer care practitioners may consider paying extra attention to these subgroups of patients. The relation between fatigue and work ability needs further research, as well as effective interventions to reduce fatigue and improve HRQL and functional performance, for example, work ability.

**Appendix 1.** Questions in the Questionnaire Regarding Fatigue, Daily and Physical Activity, Anxiety, and Depressed Mood.

Questions	Answering alternatives										
1. Have you felt an extreme tiredness that has been difficult to relieve by resting, during the past week?	Never										All the time
	0	1	2	3	4	5	6	7	8	9	10
2. How large amounts of your usual daily activities have you been able to perform during the past week?	1 = I have not managed any of my daily activities										
	2 = I have managed few (less than 1/5) of my daily activities										
	3 = I have managed less than half of my daily activities										
	4 = I have managed approximately half of my daily activities										
	5 = I have managed more than half of my daily activities										
	6 = I have managed most (more than 4/5) of my daily activities										
	7 = I have managed all my daily activities										
3. How much time do you spend at a level that makes you short winded, for example, running, fitness class or ball games?^a^	1 = 0 min										
	2 = Less than 30 min										
	3 = 31-60 min (0.5-1 h)										
	4 = 61-90 min (1-1.5 h)										
	5 = 91-120 min (1.5-2 h)										
	6 = More than 120 min (2 h)										
4. How much time are you physically active in ways that are not exercise, for example, walks, bicycling, or gardening? Add together all activities lasting at least 10 min.^a^	1 = 0 min, no time										
	2 = Less than 30 min										
	3 = 31-60 min (0.5-1 h)										
	4 = 61-90 min (1-1.5 h)										
	5 = 91-150 min (1.5-2.5 h)										
	6 = 151-300 min (2.5-5 h)										
	7 = More than 300 min (5 h)										
5. Have you experienced anxious mood during the past week?	Never										All the time
	0	1	2	3	4	5	6				7
6. Have you experienced depressed mood during the past week?	Never										All the time
	0	1	2	3	4	5	6				7

a These questions were answered regarding a regular week prior to cancer and regarding the past week of radiotherapy.

**Appendix 2.** Symptom Occurrence Reported in the MSAS.

Symptom, *n* (%)	Total study group of patients receiving cancer-related radiotherapy, n = 458
Lack of energy, n = 443	301 (68)
Problems with sexual interest or activity, n = 435	223 (51)
Feeling drowsy, n = 440	223 (51)
Difficulty sleeping, n = 442	220 (50)
Pain, n = 445	220 (49)
Sweats, n = 443	214 (48)
Worrying, n = 439	193 (44)
Feeling sad, n = 444	186 (42)
Dry mouth, n = 443	180 (41)
Difficulty concentrating, n = 443	178 (40)
Shortness of breath, n = 439	154 (35)
Diarrhea, n = 440	152 (34)
Nausea, n = 443	152 (34)
Numbness/tingling in hands/feet, n = 440	152 (34)
Cough, n = 443	145 (33)
Feeling irritable, n = 438	141 (32)
Lack of appetite, n = 440	139 (32)
Feeling nervous, n = 444	134 (30)
Problems with urination, n = 443	123 (28)
Dizziness, n = 442	123 (28)
Feeling bloated, n = 444	120 (27)
Change in skin, n = 444	117 (26)
Itching, n = 442	117 (26)
“I don′t look like myself,” n = 446	107 (24)
Change in the way food tastes, n = 446	103 (23)
Weight loss, n = 446	89 (20)
Difficulty swallowing, n = 442	86 (20)
Constipation, n = 443	74 (17)
Swelling in arms or legs, n = 446	60 (14)
Hair loss, n = 445	56 (13)
Vomiting, n = 443	53 (12)
Mouth sores, n = 445	51 (12)

*n* of patients delivering data is presented regarding the 32 symptoms asked for. Data presented as n (%). The MSAS assesses the occurrence, intensity, and distress of 32 symptoms and the frequency of 24 symptoms.^42^

MSAS, Memorial Symptom Assessment Scale.
